# Dr. Couper, on Generation

**Published:** 1801-05

**Authors:** 


### *To the Editors of the Medical and Phyfical Journal.*

Gentlemen,

I Live in a remote part of the country, where accefs to libraries, either public or private, is not very frequent; and the price, the fhamelefs price, of new books now driving them out out of the reach of ordinary purfes, I have been obliged for years paft to fatisfy my literary curiofity chiefly with Reviews, Journals, and the like, which I fear, for the fame reafon, will foon be out of my reach too. A year or two ago I faw Speculations on Generation mentioned in the Reviews with fome praife; and as the book was faid to overthrow the generally received theory, and to point out a new one, and as the price did not put it out of my reach, I purchafed a copy of it. It was written by a Dr. Couper; and though I receive the ftatements and conjectures of medical men with much hefitation and allowance, the Doctor's ingenuity, at leaft, muft be admitted. He fets off with a furious attack on the long eftablifhed theory; and I cannot help going along with fome of the Reviewers, who unequivocally admit his complete fuccefs in overthrowing it; and he muft be a hardy writer or Profeffor, indeed, who, after fuch an elucidation, will perfevere in promulgating the old doctrines.

With refpect to the new idea of generation, which Doctor Couper has thrown out, but which, to do him juftice, he does not affect to have by any means eftablifhed, it wears as plaufible a face as moft theories; and, as he fays, may in time, and in better hands, be as well worth the acknowledging as the beft of them. He alleges, that the femen is abforbed by the vagina, which is peculiarly conftructed for this very purpofe; that it is thus thrown into the general circulation, impregnating the whole mafs; the refult of which, like other functions for other ends, is determined to the ovaria. This he confiders probable from the powerful effects of the femen reforbed by the male; from its effects on the female when impregnation does not take place, as well as when it has taken place; and from a multitude of appearances and circumftances which I fhall not occupy more of your room with; but which, whether the theory be followed up and become ufelefs, or whether it fhall fink into obfcurity, like many a theory before it, will evince no little attention and ingenuity in the author.

Why I have taken upon me to mention this book in your ufeful work is, neither to attack nor defend it, but merely to obferve that, fince its publication, I have feen and heard of different theories of generation, the authors of which would not have been lofers by a perufal of it.

I am, your's, &c.

AMICUS.

